# Dr. Deiters on the Use of Cubebs in Infantile Enuresis

**Published:** 1855-01

**Authors:** 


Dr. DEITERS *on the Use of Cubebs in Infantile Enuresis.*—This author has found cubebs more effectual than any other remedy in curing the incontinence of urine so common among children. This complaint may depend upon atony of the bladder, or on the presence of intestinal worms. In the former case the cubebs acts as a tonic, in the latter as a valuable anthelmintic. The medicine requires to be given in considerable doses; two pinches (*i. e.* a few grains or *Zwei Messerspitzvoll*) for infants, and a half a tea spoonful twice or thrice daily for children of a somewhat more advanced age. Its effect is speedy and permanent; and although occasionally it happens that during its administration the incontinence returns at periodical or irregular intervals, these recurrences gradually become less frequent, and eventually disappear altogether. To effect a radical cure, the author has often found it necessary to continue its use for a period of from three to eight weeks, and he has never observed any injurious effects from its administration.

Deiters observes that he has found the same remedy most efficacious in checking nocturnal emissions in case of spermatorrhea.—*Edinburgh Monthly Journal, from Preus. Verein. Zeitung.*

